# Evaluation of Setting Time, Flowability, Film Thickness, and Radiopacity of Experimental Monocalcium Silicate-Based Root Canal Sealers

**DOI:** 10.1155/2024/8541653

**Published:** 2024-04-20

**Authors:** Sukanya Juntha, Pakit Tungsawat, Ninnita Wongwatanasanti, Warattama Suksaphar, Siripat Lertnantapanya

**Affiliations:** College of Dental Medicine, Rangsit University, Bangkok, Pathum Thani, Thailand

## Abstract

**Introduction:**

This study aimed to evaluate the efficacy of a formulation of premixed calcium silicate-based sealer (CSBS) with monocalcium silicate (Mono-CS) as the main component. Its properties were compared with those of a control group (iRoot SP) according to ISO 6876/2012 standards for root canal sealers.

**Materials and Methods:**

The CSBS formulation consisted of two components (powder and liquid). The powder was a mixture of Mono-CS, a radiopacifier, and a thickening agent, and the liquid components were nonaqueous liquid agent and setting accelerator. Three formulation groups with different powder–liquid ratios were prepared: group A, 2 : 1; group B, 3 : 1; and group C, 2 : 1, which also contained calcium chloride as a setting accelerator. The setting time, flow rate, film thickness, and radiopacity of the three CSBS groups and the control group were evaluated and compared. Each test was repeated five times for each group.

**Results:**

The minimum values of setting time (i.e., working time, initial setting time, and final setting time) were ranked in order of significance as group B, the control group, group C, and group A. The control group had the lowest film thickness at 20 *μ*m, with a nonsignificant difference to group C. The flow rates in group A, group C, and the control group were >20 mm. Furthermore, the experimental groups showed a similar amount of radiopacity as the control group (*p*  > 0.05).

**Conclusion:**

Mono-CS and calcium chloride can be used in the formulation of root canal sealers, and their properties, including working time, initial setting time, final setting time, flow rate, film thickness, and radiopacity, are consistent with those of iRoot SP and ISO 6876/2012 standards.

## 1. Introduction

Root canal therapy aims to treat and prevent apical periodontitis. Thus, bacterial reduction or elimination using both chemomechanical preparations and intracanal dressings is essential [1]. Additionally, the root canal filling material is an important factor. It is used in the obturation of the root canal system to achieve a hermetic or fluid-tight seal throughout the canal, including the apical foramen, irregularities in the canals, and lateral and accessory canals, and to fill the space between the root canal wall and the gutta-percha [1, 2]. Therefore, sealers play an important role in preventing leakage and invasion of periapical tissue by residual bacteria and promoting the healing of periapical lesions [3]. Grossmann suggested that an ideal root canal sealer should have excellent sealing ability and slow setting time to ensure sufficient working time, dimensional stability, and biocompatibility. It should be soluble in solvents if it is necessary to remove the root canal filling [4]. In the past decade, bioceramic materials based on various calcium silicates have been introduced to improve root canal sealing and hydroxyapatite formation to create a hermetic seal between the root canal dentin and sealer. These characteristics render it a promising option for clinicians [5].

The first endodontic sealer of this type introduced in 2007 was iRoot SP (Innovative Bioceramix, Vancouver, Canada). It is an injectable premixed calcium silicate-based sealer (CSBS) composed of zirconium oxide, tricalcium silicates, dicalcium silicates, calcium phosphate monobasic, calcium hydroxide, fillers, and thickening agents [6]. iRoot SP can be considered for clinical use because of its favorable physical and biological properties such as nontoxicity, biocompatibility, hydrophilicity, use of intracanal moisture from the dentinal tubules to initiate and complete its setting reaction, highly alkaline pH, dimensional stability, and lack of shrinkage [7]. With respect to the favorable characteristics of CSBS, other products based on bioceramic materials such as tricalcium silicates and dicalcium silicates have been increasingly introduced [8].

Because they are mainly composed of tricalcium silicate and dicalcium silicate, CSBSs are considered bioactive bioceramic materials. They release Si and Ca ions, which have an important role in stimulating cell proliferation and differentiation [9]. Tricalcium silicate is the most important component and interacts quickly with water to create a variety of polymorphic crystalline phases depending on temperature, composition, and impurities [10, 11]. The hydration reaction primarily influences the setting and development of early strength [11]. Notably, dicalcium silicate dissolves far more slowly than tricalcium silicate, which contributes to later strength. Dicalcium silicate cement has a strong apatite-forming activity and shows minimal deterioration under acidic conditions [11]. Several studies have investigated the parameters of both types of calcium silicate [11].

Monocalcium silicate (Mono-CS) is a bioceramic material that has been widely used for various biomedical applications owing to its favorable bioactive properties, such as good biocompatibility, antibacterial activity, induction of high pH conditions, and promotion of cell differentiation and tissue regeneration [12, 13]. The use of porous *β*-calcium silicate (*β*-CaSiO_3_) in rabbit calvarial defects stimulated bone regeneration and reparative hard tissue [14]. In vitro cell culture studies revealed that calcium silicate releases Si and Ca ions, suggesting that it may have the ability to support cell attachment and induce tissue regeneration and may be appropriate for orthopedic and dental applications [15]. Thus, it has gained attention in the field of dentistry and has been comprehensively investigated as a pulp-capping agent and root-end filling material [16–18]. Recently, Mono-CS was added to glass ionomer cement (GIC) to improve both bioactivity and biocompatibility. Chaisinghanuae et al. [19] investigated the cytotoxicity of GIC containing Mono-CS and compared it with White ProRoot® and Ketac Molar in human pulp cells and found that it showed the same level of cytotoxicity [19]. Similarly, Thanapornpun et al. [16] investigated the biocompatibility and apatite formation of GIC with 50 wt% Mono-CS and reported good biocompatibility with osteoblast cells and induction of apatite formation [16].

However, there are significant concerns regarding the hardness of calcium silicate materials [7]. For example, the compressive strength of iRoot SP can reach 8.58 MPa because of its hardness upon setting, which can affect its retreatability [7, 20]. However, Mono-CS shows similar compressive strength as cancellous bone, with the value ranging from 0.2 to 4 MPa [21]. Therefore, Mono-CS, when used as the main component in root canal sealers, might facilitate endodontic retreatments while maintaining biocompactibility and bioactivity. Nonetheless, clinical validation is required.

There are no studies that have investigated the effect of Mono-CS as the main component of root canal sealers. The properties of root canal sealers should first be investigated in a laboratory settings because they affects the quality of root canal obturation [3].

Therefore, this study aimed to evaluate formulations of premixed Mono-CS-based sealers. The physical properties specified by ISO 6876/2012 (i.e., initial setting time, final setting time, working time, flow rate, film thickness, and radiopacity) were observed and compared with those of the commercial sealer iRoot SP. The null hypothesis was that the newly formulated Mono-CS-based sealer possesses adequate physical properties comparable to those of iRoot SP sealers because it is mainly contributed from calcium silicate material, nonaqueous liquid agent, setting accelerator, and radiopacifier.

## 2. Materials and Methods

iRoot SP (Innovative Bioceramix, Vancouver, Canada) was used as the control group, and three new formulas test sealers were used as the experimental groups. The physical properties, including working time, initial and final setting times, flow rate, film thickness, and radiopacity, were examined according to ISO 6876/2012 specifications, with five samples prepared for each group. The iRoot SP sealer was used according to the manufacturer's instructions.

### 2.1. Preparation of Materials and Samples

Table 1 shows that the main component of the experimental samples was Mono-CS (form: powder, reactionary suitable: core calcium, white color, particle size: 7.0–10.0 *μ*m, pH 9.5–11.5) and the other components were hydroxypropyl methylcellulose (form: powder, 86 kDa mol weight, white color, viscosity 2,600–5,600 cP, 2% in H_2_O (20°C) (L), zirconium oxide (form: powder, particle size: <10 *μ*m, white color), polyethylene glycol-400 (form: viscous liquid, colorless, 400 mol weight, pH 4.5–7.5, viscosity 7.3 cSt (210°F) (L), and calcium chloride (form: viscous liquid, colorless, ∼1 M in H_2_O, pH 4–6) (Sigma–Aldrich Pte. Ltd., Singapore).

The sealer tested in each group was prepared by mixing purified Mono-CS with the other powdered components using the geometric dilution method. They were then mixed with the liquid component according to the defined powder–liquid ratio.

### 2.2. Examination of the Physical Properties According to ISO 6876/2012 Specifications

#### 2.2.1. Flow Rate

A volume of 0.05 ± 0.005 mL of the test sealer was placed on the center of a glass plate (40 mm × 40 mm × 5 mm) using a 1-mL syringe. A second glass was placed centrally on top of the sealer, and an additional mass of 100 g was placed on the plate to total 120 ± 2 g after 3 min. Ten minutes after placing the sealer on the glass plate, the glass plate and load were removed. The minimum and maximum diameters of the sample disks were measured using digital calipers with an accuracy of up to 0.01 mm. If the disks were not uniformly circular, or the maximum and minimum diameters were not within 1 mm, the test was repeated. If the diameters were within 1 mm of each other, the mean of the two diameters was calculated. The test was repeated five times for each group and the mean value was considered as the flow rate of the material.

#### 2.2.2. Setting Time

Plaster molds were stored in cabinets at 37 ± 1°C and 95% humidity for 24 hr. The test sealers were inserted in plaster molds of 10 mm diameter and 1 mm depth on a flat glass plate 1 mm thickness after 120 ± 10 s from the end of mixing. This mold was placed on a metal block measuring at least 8 mm × 20 mm × 10 mm and stored in the cabinet at 37 ± 1°C and 95% humidity. As the setting time approached, a Gilmore-type indenter and flat-end needle were carefully lowered vertically onto the horizontal surface of the sealer. Sealers were considered set if the needle did not leave a visible indentation when gently lowered onto the surface of the material. The setting time was recorded as the end of mixing until the sealer was set. This test was conducted five times for each group, and the calculated mean value was considered the setting time.

#### 2.2.3. Working Time

ISO 6876/2012 does not specifically require measurement of the working time of the tested sealers. Therefore, as per the study by Lyu et al. [22], the working time in this study was assessed by following the same procedure as that used for the flow test. The time interval was recorded as the working time at which the mean diameter decreased to 17 mm [22].

#### 2.2.4. Film Thickness

Sealers were placed between two glass plates with contact surface areas of ∼200 × 25 mm^2^. After 180 ± 5 s since the glass plates were placed, a load of 150 N was applied vertically on top of the glass plate. After 10 min since the placement, the thickness of the combined glass plates and sealer was measured using a micrometer of accuracy up to 1 *μ*m. The total thickness of the two glass plates in contact was measured before the sealer was dispensed between them. The film thickness of the sealer was determined as the difference between the thicknesses of the glass plates with and without sealer. Five measurements were performed for each group, and the mean value was calculated as the film thickness.

#### 2.2.5. Radiopacity

The sealers were placed in stainless steel molds of 10 mm diameter and 1 mm thickness. The mold was placed centrally on a glass plate, and a second glass plate was placed on top. The specimen was placed at the center of a size 4 X-ray film, adjacent to the aluminum step wedge with a thickness of 5.0 mm in equally sized steps of 1 mm. The specimen, step wedge, and X-ray film were irradiated at 60 kV and 8 mA for 0.016 ms (X-MIND DC, Acteon, Olgiate Olona, Italy) at a fixed distance of 300 mm between the X-ray tube and the target plate. The X-ray film was scanned using an imaging-plate scanner (Gendex DenOptix QST), and the radiographic image was saved as a “.jpg” file. The gray values of the tested sealer materials and the aluminum step wedge in the radiographic images were measured using the ImageJ program (National Institutes of Health, Bethesda, MD, USA). The radiopacity of the specimen is expressed in millimeters of aluminum thickness (mmAl). The means and standard deviations were calculated for radiopacity.

## 3. Statistical Analysis

Five samples were collected from each group to compare the six properties of the three proposed formulas with those of the iRoot SP sealer. The median difference in the six properties among the three groups of tested sealers was compared using the Kruskal–Wallis test, and the Mann–Whitney *U* test was used as a post-hoc test to compare pairwise differences between the groups. Finally, descriptive statistics were used to investigate the proportion of qualified samples according to the ISO cutoff. SPSS version 25.0 was used to analyze the data.

## 4. Results

Table 2 shows comparison of the physical properties among the four groups of sealers including initial setting time, final setting time, working time, flow rate, film thickness, and radiopacity.

The time-dependent properties (initial, final, and working times) differed significantly in all pairwise comparisons of the sealers. All recorded times were the lowest in group B, followed by iRoot SP, group C, and group A. The initial setting time was <24 hr in groups B and C and iRoot SP, whereas that in group A was >24 hr. The final setting time was 73 hr in groups B, C, and iRoot SP, whereas that in group A was >160 hr. Finally, the working time was within 2 hr in groups B, C, and iRoot SP, whereas it was >24 hr in group A.

Table 2 shows the similarities in other properties between the test sealers and the iRoot SP sealer. Most sealers were consistent with the iRoot SP sealer in terms of radiopacity (*p*  > 0.05). All the tested sealers demonstrated radiopacity >4 mmAl, which corresponds to that of the iRoot SP and the ISO 6876/2012 recommendation (≥3 mmAl). iRoot SP had the lowest film thickness at 20 *μ*m with nonsignificant difference from group C (*p*  > 0.05). On the other hand, the group B sealer had the highest film thickness, which was not consistent with the ISO 6876/2012 recommendation (≤50 *μ*m). In the flow rate test, the flow of all the tested sealers was >20 mm, except that of the group B sealer (17.25 mm), which was significantly lower than that of the other three groups. However, it was consistent with the ISO 6876/2012 recommendation (≥17 mm).

## 5. Discussion

While endodontic sealers are being continuously developed, it is important for clinicians to understand their physicochemical properties for appropriate application because the effectiveness of sealers primarily depends on their chemical composition and proportion [3]. Currently, CSBS with tricalcium silicate and dicalcium silicate as the main components are widely used because of their excellent physicochemical properties, biocompatibility, and bioactivity, as well as their ability to promote hydroxyapatite formation [23]. Considering these advantages, it is one of the best sealing materials in dentistry. The currently popular commercial sealer iRoot SP (Innovative Bioceramix, Inc., Vancouver, Canada) was launched in the market under the commercial name EndoSequence BC Sealer (Brasseler USA, Savannah, GA, USA) and mainly comprises tricalcium silicate, dicalcium silicate, and calcium phosphate monobasic [24].

However, in recent years, Mono-CS has been widely used owing to its ability to induce in vivo osseointegration and promote bone repair and tissue regeneration [13, 14, 25]. It has been increasingly investigated in preclinical dentistry studies for its role as a root-end filling material or pulp-capping agent owing to its biocompatibility and bioactive properties [16–18]. To date, no study has investigated the effects of Mono-CS as the main component of root canal filling materials. Therefore, the development of a formulation for premixed monocalcium silicate-based sealers is proposed. This study showed that the initial setting time, final setting time, working time, flow rate, film thickness, and radiopacity of the tested sealers were consistent with that of the iRoot SP and ISO 6876/2012 recommendations.

The composition, particle size, shear rate, and temperature are the main factors affecting the properties of flow and film thickness of sealers. Sealer flow is an important factor affecting the outcome of the final root canal filling. Acceptable flow within the working time is important for any endodontic sealer to reach and seal the apical foramen, irregular areas, and lateral canals. However, excessive flow increases the probability of sealer extrusion into the periodontal tissues [3].

According to ISO 6876/2012, the flow should be >17 mm. In our study, the flow of the group A sealer was 20.21 mm, which was consistent with the findings of Qu et al. [26] and Zhou et al. [3] who reported a flow of 22.9 and 23.1 mm, respectively, for iRoot SP [3, 26]. Because the flow of the group B sealer was 17.25 mm, the group A sealer was more similar to iRoot SP than the group B sealer. Nevertheless, the film thickness of the group B sealer was greater than that of the group A sealer, iRoot SP, and the standard ISO 6876/2012 recommendation; this high degree of viscosity resulted in faster setting time of the group B sealer than that of the other groups. The difference in the powder–liquid ratios of groups A and B (2 : 1 and 3 : 1, respectively) might imply that the quantity of powder content in group B, such as Mono-CS, zirconium oxide, and hydroxypropyl methylcellulose (HPMC), was greater than that of the liquid content (polyethylene glycol (PEG) 400), thus contributing to increased viscosity.

Although the physical properties of the group A sealer were satisfactory, the setting time was longer than that required for sufficient work. Thus, the composition of the group A sealer was refined by adding calcium chloride as an accelerator to increase the hydration rate and decrease the setting time. Calcium chloride is one of the most effective accelerators of hydration and setting used in Portland cement pastes [27]. Calcium chloride has also been used as the reaction accelerator in BioRoot RCS (Septodont, St. Maur-des-Fossés, France) [28]. Wang et al. [29] investigated the setting time, compressive strength, and bioactivity of tricalcium silicate combined with various concentrations of calcium chloride. They showed that once calcium chloride entered the pores of tricalcium silicate particles, the chloride ions bound to the calcium silicate–hydrate gel to form a calcium oxychloride complex. Thus, the sizes of the macropores and micropores decrease when the calcium chloride concentration approaches 10 wt%, leading to an acceleration of the setting time; however, the diameter increases again when the calcium chloride content reaches 15 wt%, whereas the setting time does not change. Hence, the compressive strength increases and reaches a maximum value with 10 wt% calcium chloride and decreases at a concentration higher than the threshold. Based on scanning electron microscopic analysis, a bone-like apatite layer was formed on the surface of the cement in the simulated body fluid for all concentrations of calcium chloride [29].

Therefore, the group C sealer formulation was developed to enhance the efficacy of the group A sealer. This was achieved by maintaining the powder quantity and reducing the quantity of the PEG 400 with the addition of aqueous calcium chloride instead of reducing the setting time. Thus, the addition of 10 wt% calcium chloride accelerated the setting time and did not interfere with the sealer during apatite formation. The ideal setting time of a root canal sealer should permit sufficient working time because a slow setting time can cause tissue irritation with a certain degree of toxicity until the sealer finally sets [30]. With the modification made to the group C sealer, its setting time had a similar range as that of iRoot SP and was faster than that of the group A sealer. The initial setting time was 6 hr, whereas that of the group A sealer was >24 hr. The final setting time of the group C sealer was within 73 hr, whereas that of the group A sealer was >160 hr. These findings show that the characteristics of the group C sealer are consistent with those reported by Chen et al. [5], where the initial setting time of Endosequence BC sealer was 4.7 hr and the final setting time was 72.7 hr at 37°C [5]. Similarly, the setting time of iRoot SP was previously reported by Qu et al. [26] to be 4 hr.

These findings cumulatively indicate that the group C sealer had a faster setting time after the addition of 10 wt% of calcium chloride to its liquid component, which served as a catalyst for the hydration of Mono-CS and resulted in the formation of small fibrous crystals during hydration. Thus, crystallization occurs rapidly because calcium chloride can penetrate the pores of Mono-CS and reduce the mean pore diameter, thus accelerating the hydration reaction [29]. Furthermore, it is water-soluble and can be integrated with Mono-CS and other components, which can accelerate the setting time and not affect the other properties negatively. Therefore, the flow of the group C sealer remained at >20 mm and was not clinically different from that of iRoot SP. In addition, the film thickness of the group C sealer was not significantly different from that of iRoot SP (*p*  > 0.05). However, the group B sealer had the highest film thickness among the tested sealers, which was not consistent with the ISO 6876/2012 recommendation (≤50 *μ*m), as a higher degree of film thickness can affect the sealing ability of root canal sealers [5].

For the flow and the film thickness, the influencing component is the liquid component, PEG 400, which has biomedical applications such as drug delivery. Its low molecular weight makes it less viscous. This hydrophilic property contributes to the water-soluble polymer property; that is, it is a nonaqueous but water-miscible liquid that functions as a cosolvent capable of reducing the polarity of Mono-CS and water and decreasing the resistance among the particles. Therefore, these components can integrate significantly and enhance particle solubility and flow [31]. To prevent the sealers from becoming watery, HPMC, a thickening agent, is added to increase the gelling ability to form viscous solutions, which improves the washout resistance. Furthermore, the addition of HPMC to the cement increases the cohesiveness and plasticity of the material, making it easier to manipulate [11, 31]. Xu et al. [31] suggested the use of PEG 400 as the nonaqueous liquid component and HPMC as the thickening agent when producing premixed calcium phosphate cement (CPC). They found that CPC could be transformed into a premixed state that did not harden during storage or in a syringe. The paste hardened only after contact with physiological fluids. Moreover, PEG 400 and HPMC do not negatively affect osteoblast viability [31].

Root canal sealers should be adequately radiopaque to distinguish them from nearby anatomical structures, thus facilitating the assessment of root filling quality on radiographic examination [30]. Zirconium oxide is used as a radiopacifier in root canal sealers such as iRoot SP because of its good biocompatibility, nondiscoloration, inert properties, nonleaching material, and ISO standard contrast media [3]. Camilleri et al. [32] investigated the effect of adding 20 wt% zirconium oxide to tricalcium silicate cement. They found that the cement was biocompatible, allowed apatite formation on the cement surface, and presented a preferred radiopacity of >3 mmAl [32]. Furthermore, Li et al. [33] found that adding 50 wt% zirconium oxide to tricalcium silicate cement was biocompatible with tissues and did not induce cytotoxicity in dental pulp fibroblasts [33]. However, the proportion of radiopacifiers should be optimized without altering the properties of the main material. If the radiopacifier content is increased to enhance radiopacity, the proportion of the main active ingredients would have to be reduced, thus compromising material properties, such as push-out strength and compressive strength [34].

The radiopacity in this study was consistent with ISO recommendations by using 30 wt% zirconium oxide and it does not have any negative effects on the main components. Thus, the tested sealers demonstrated radiopacity >4 mmAl, which was consistent with the ISO 6876/2012 recommendation (≥3 mmAl). The radiopacity of the group C sealer did not differ significantly from that of iRoot SP (*p* < 0.05) and was consistent with that of the Endosequence BC sealer (4.7 mmAl) reported by Chen et al. [5].

In this study, premixed Mono-CS-based sealers of group C were developed by adding other important components, such as zirconium oxide as a radiopacifier, calcium chloride as the accelerator, PEG 400 as the filler, and HPMC as the thickening agent. With these parameters, it fulfilled the ISO 6876/2012 standards with physical characteristics (i.e., initial setting time, final setting time, working time, flow rate, film thickness, and radiopacity) similar to those of iRoot SP.

In this study, 65 wt% Mono-CS was added to increase the quantity of the main component over the other substances to ensure favorable bioactivity and deposition of apatite formation. However, Mono-CS is brittle with a porous surface and poor handling properties [18]. This can be refined by adding other substances to improve their properties and stability. Sangsawatpong et al. [18] also showed that apatite formation occurred with 50 wt% Mono-CS combined with GIC in 7 days, with a similarity between the calcium–phosphorus and hydroxyapatite ratios [18]. Additionally, Thanapornpun et al. [16] showed that 50 wt% Mono-CS with GIC had good biocompatibility with osteoblasts and could induce apatite formation [16]. The main reason for using 50 wt% Mono-CS with GIC is the synergistic effect between them. However, in our study, we took advantage of the bioactivity of Mono-CS by adding more than 50 wt% Mono-CS to the other components.

A limitation of this study is that the results from the preclinical study evaluated the initial setting time, final setting time, working time, flow rate, film thickness, and radiopacity of premixed Mono-CS-based sealers according to ISO 6876/2012 standards. Additional research should be conducted on all other aspects in both in vitro and in vivo studies (e.g., cytotoxicity, calcium release, pH change, solubility, leakage, and retreatability) before employing it in clinical practice.

## 6. Conclusion

The premixed CSBS formulation of the group C sealer with Mono-CS as the main component and calcium chloride as the setting accelerator has properties consistent with the ISO 6876/2012 specifications with respect to the initial setting time (6 hr), final setting time (73 hr), flow rate (21.44 mm), film thickness (31 *μ*m), and radiopacity (4.34 mmAl). Moreover, the properties of the group C sealer are similar to those of iRoot SP. Therefore, the group C sealer has the potential for further development in future studies.

## Figures and Tables

**Table 1:** Preparation of the study materials.

Experimental groups	Composition		Powder–liquid ratio
	Powder	Liquid	
Group A: Regimen 1	(i) Monocalcium silicate, 65 wt% (ii) Hydroxypropyl methylcellulose, 5 wt% (iii) Zirconium oxide, 30 wt%	(i) Polyethylene glycol 400	2 : 1

Group B: Regimen 2	(i) Monocalcium silicate, 65 wt% (ii) Hydroxypropyl methylcellulose, 5 wt% (iii) Zirconium oxide, 30 wt%	(i) Polyethylene glycol 400	3 : 1

Group C: Regimen 3	(i) Monocalcium silicate, 65 wt% (ii) Hydroxypropyl methylcellulose, 5 wt% (iii) Zirconium oxide, 30 wt%	(i) Polyethylene glycol 400 (ii) Calcium chloride, 10 wt%	2 : 1

**Table 2:** Summary of the statistical analyses of the physical properties of the sealers.

Physical properties	Median (Q1–Q3)			
	Group A	Group B	Group C	iRoot SP
Initial setting time (hr)	31.15^a^ (31.15–31.15)	4.36^b^ (4.37–4.38)	6.04^c^ (6.03–6.04)	4.50^d^ (4.50–4.51)

Final setting time (hr)	167.07^a^ (167.05–167.07)	71.31^b^ (7.30–71.33)	73.14^c^ (73.12–73.15)	72.54^d^ (72.53–72.55)

Working time (hr)	25.34^a^ (25.33–25.35)	0.4525^b^ (0.452–0.4525)	2.05^c^ (2.05–2.06)	0.552^d^ (0.542–0.553)

Film thickness (*μ*m)	43^a^ (41–44)	58^b^ (52–67)	31^c^ (30–31)	20^c^ (15–30)

Radiopacity (mmAl)	4.043^a^ (3.75–4.23)	4.71^b^ (4.37–4.23)	4.34^a,b,c^ (4.27–4.39)	4.38^a,b,c^ (4.18–4.71)

Flow rate (mm)	20.21^a,d^ (20.12–20.48)	17.25^b^ (17.06–17.57)	21.44^a^ (21.32–21.48)	20.29^d^ (20.06–20.66)

^a–d^Different letters on the same line indicate statistically significant differences between experimental groups (*p* < 0.05).

## Data Availability

The datasets used and/or analyzed during the current study are available from the first author upon request.

## Abbreviations

CSBS: Calcium silicate-based sealers
Mono-CS: Monocalcium silicate
GIC: Glass ionomer cement
HPMC: Hydroxypropyl methylcellulose
PEG 400: Polyethylene glycol (molecular weight 400)
CPC: Calcium phosphate cement.
